# Evaluation of cellular alterations and inflammatory profile of mesothelial cells and/or neoplastic cells exposed to talc used for pleurodesis

**DOI:** 10.18632/oncotarget.27750

**Published:** 2020-10-13

**Authors:** Milena Marques Pagliarelli Acencio, Bruna Rocha Silva, Lisete Ribeiro Teixeira, Vanessa Adélia Alvarenga, Carlos Sérgio Rocha Silva, Aline Graças Pereira da Silva, Vera Luiza Capelozzi, Evaldo Marchi

**Affiliations:** ^1^Laboratorio de Pleura-Divisao de Pneumologia, Instituto do Coracao, Hospital das Clinicas HCFMUSP, Faculdade de Medicina, Universidade de São Paulo, São Paulo, Brazil; ^2^Department of Pathology, Hospital das Clinicas HCFMUSP, Faculdade de Medicina, Universidade de São Paulo, São Paulo, Brazil; ^3^Medical College of Jundiai, São Paulo, Brazil

**Keywords:** pleural mesothelial cells, talc, pleurodesis, malignant pleural effusion, cellular alterations

## Abstract

Introdution: To determine the role of Pleural Mesothelial Cells (PMC) and/or Neoplasic Cells (NC) in the initiation and regulation of acute inflammatory response after exposure to talc for evaluating inflammatory mediators and cellular alterations.

Materials and Methods: PMC cultures, human lung (A549) and breast (MCF7) adenocarcinoma cells were divided in 5 groups: 100% PMC, 100% NC, 25% PMC + 75% NC, 50% of each type and 75% PMC + 25% NC. All groups were exposed to talc and measured IL-6, IL-1β, IL-10, TNF-α, TNFRI, pH, LDH, apoptosis and necrosis. Statistical Analysis: One-way Anova.

Results: High IL-6, IL-1β and TNFRI levels were found in PMC and NC exposed to talc. IL-6 was higher at the points of more confluence of PMC. The highest levels of IL-1β and TNFRI were found in mixed cultures. In pure cultures TNFRI was higher in A549 followed by PMC and MCF7. LDH was higher in A549 than PMC. The lowest pH was found in 100% NC. All cell line exposed to talc reduced viability and increased necrosis. Apoptotic cells exposed to talc were higher in pure cultures of NC than in PMC. Mixed cultures of PMC and A549 showed lower levels of apoptosis in cultures with more NC.

Conclusions: PMC after talc exposure participates in the inflammatory process contributing to production of molecular mediators, necessary for effective pleurodesis. Talc acted in NC causing higher rates of apoptosis, contributing in a modest way to tumoral decrease. Different types of tumor cells may respond differently to exposure to talc.

## INTRODUCTION

Metastatic neoplasms are the most common type of pleural neoplastic disease and the principal primary sites are lung, breast, stomach and ovary [1]. Adenocarcinoma is the most common cellular type, and the majority of patients present pleural effusion, whose treatment is only palliative [2].

Several studies have demonstrated the superior efficacy of talc over other sclerosing agents, making it the preferred agent for pleurodesis according to medical research [3, 4].

Despite the wide clinical use of talc, the exact mechanisms of its action as well as its effects on cancer cells have been poorly studied. Studies have reported that pleural mesothelial coating may be the main target for the sclerosing agent to play its key role in the process of pleurodesis, including the release of various mediators such as cytokines, chemokines and growth factors among others [5].

In an experimental model of pleurodesis acute inflammatory reaction to talc was observed with an increase in pleural fluid concentrations of IL-8, VEGF and TGF-β detected after intrapleural injection of talc and noted that the mesothelial cell layer was preserved. Thus, mesothelial cells appear to participate in the response to talc and contribute to the acute inflammatory response [7, 8].

Cytokines, with multiple biological functions, are important for the initiation, perpetuation and resolution of inflammatory responses of the pleura [9]. The main cytokines responsible for this initial response are tumor necrosis factor-alpha (TNF-α) and interleukin-6 (IL- 6).

Lactic dehydrogenase (LDH) is another important inflammatory marker in cell exposure to particulate matter. LDH is located in the cytoplasm of tissue cells throughout the body, being released in the occurrence of cell damage. Almost all types of cancer as well as several other diseases can provoke high levels of LDH; in addition, its detection is extremely useful in the laboratory approach to several situations that indicate tissue injury [10].

When the tumor is in advanced stage, the presence of normal mesothelial cells is scarce; it is speculated that the response to the sclerosing agent becomes diminished and consequently the success of pleurodesis could be reduced. In addition, tumors such as malignant diffuse mesothelioma and lung carcinomas may also interfere with the outcome of pleurodesis [11].

In a previous study, it was hypothesized that patients with the mesothelium less compromised by the tumor are more likely to respond to pleurodesis successfully than in patients with extensive tumor involvement in the pleural mesothelium [6]. Some authors discuss the importance of cell death caused mainly by apoptosis in mesothelial and/or neoplastic cells leading to the success or failure of pleurodesis, or even acting to decrease the tumor [12].

The type of tumor in the pleural cavity may also affect the outcome of pleurodesis. In preliminary experimental studies it has also been suggested that talc can induce apoptosis in tumor cells and inhibit angiogenesis, thus contributing to a better control of malignant pleural effusion [12].

The hypothesis of our study is to determine the role of mesothelial and/or neoplastic cells in the initiation and regulation of the acute inflammatory response following the instillation of talc in the pleural space, evaluating cellular aspects such as apoptosis and inflammatory mediators.

## RESULTS

During the experimental protocol, PMC were isolated from transudative pleural effusions obtained via thoracentesis from five patients secondary to congestive heart failure without evidence of infection.

### Quantification of cytokines

After exposure of PMC and/or neoplastic cells (NC) to talc particles, we observed that the concentrations of IL-6, IL-1β and TNFRI were significantly higher than the cultures unexposed to talc particles (*p* < 0.001).

**Figure 1:**
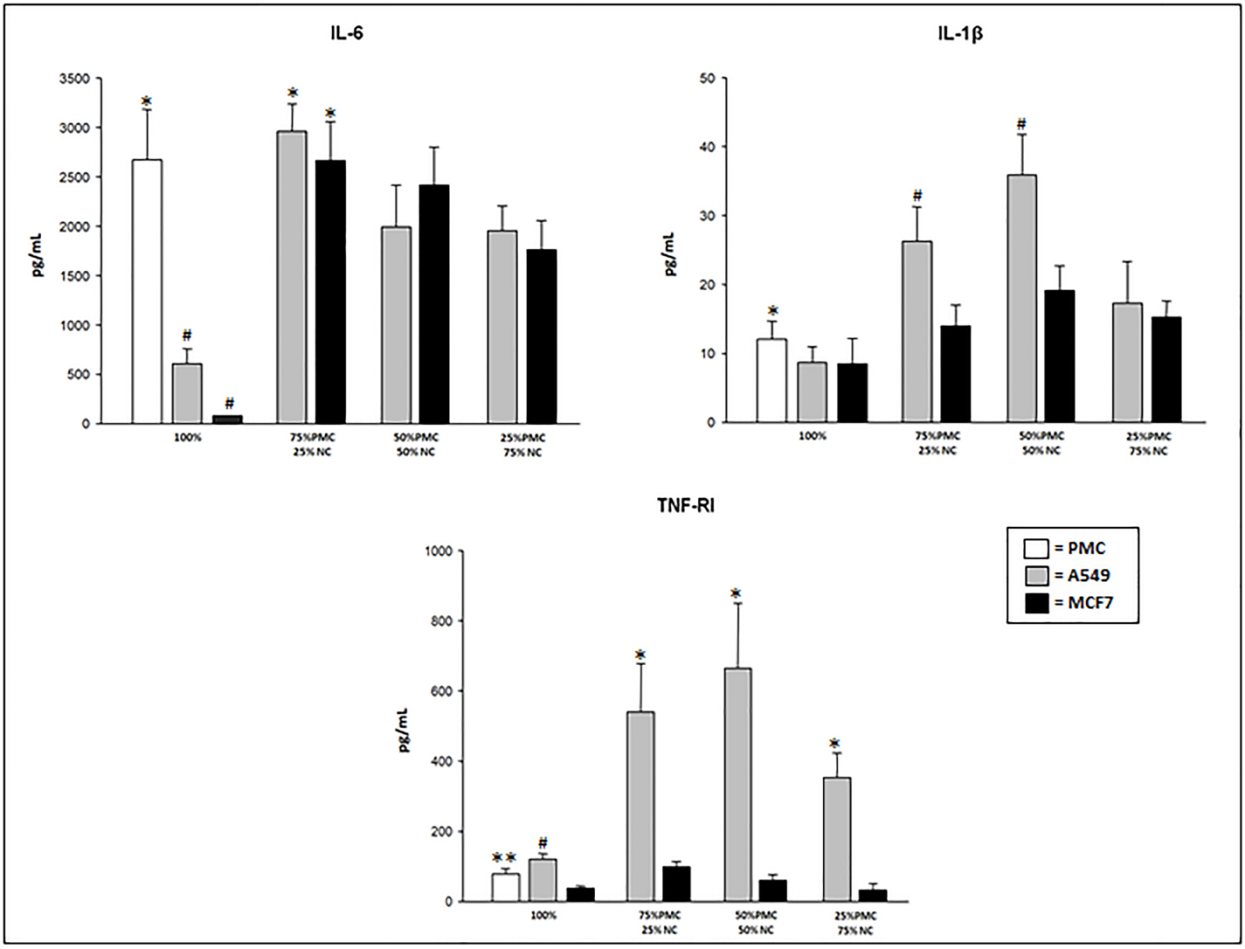
IL-6, IL-1β, and TNFRI concentrations produced by talc-exposed PMC, A549 and/or MCF7 cells after 24 hours. PMC = pleural mesothelial cells; NC = neoplastic cells. ^*^ *p* < 0.05 – IL-6 (PMC vs neoplastic cells in culture pure and 75% PMC+25%NC vs mixed cultures); IL-1β (PMC vs neoplastic cells in culture pure) TNF-RI (A549 mixed vs MCF7 mixed); ^#^ *p* < 0.001 – IL-6 (100% of A549 and MCF7 vs 100% PMC); IL-1β (75% PMC+25% NC and 50% PMC+50% PMC of A549 vs 75% PMC+25% NC and 50% PMC+50% PMC MCF7); TNFRI (100% A549 vs 100% MCF7); ^**^ *p* < 0.05–TNFRI (100% PMC vs 100% MCF7).

The IL-6 levels were significantly higher at the points with more confluence of PMC (100 and 75% of PMC). Groups with only NC (100% A549 or MCF7) presented the lowest levels of this cytokine, after exposure to talc (Figure 1).

The concentration of IL-1β was significantly higher in the group with 100% PMC (12.1 ± 2.6) than in groups with 100% A549 (8.7 ± 2.3) or 100% MCF7 (8.5 ± 1.7) after exposure to talc particles. However, highest levels were observed in the mixed cultures, mainly in the cultures with A549 cells (Figure 1).

The production of TNFRI also was higher in the mixed cultures with A549 cells exposed to talc. In the pure cultures (100% of cells), TNFRI was higher in A549 cells followed by PMC and MCF cells when exposed to talc (Figure 1). In the cultures with only PMC, A549 or MCF7 not exposed to talc particles the TNFRI levels were undetectable (< 15.6 pg/mL).

TNF-α and IL-10 levels in the supernatant of PMC and/or NC cultures showed up at less than15.6 pg/mL, undetectable by the proposed measurement technique.

### LDH and pH measurement

**Figure 2:**
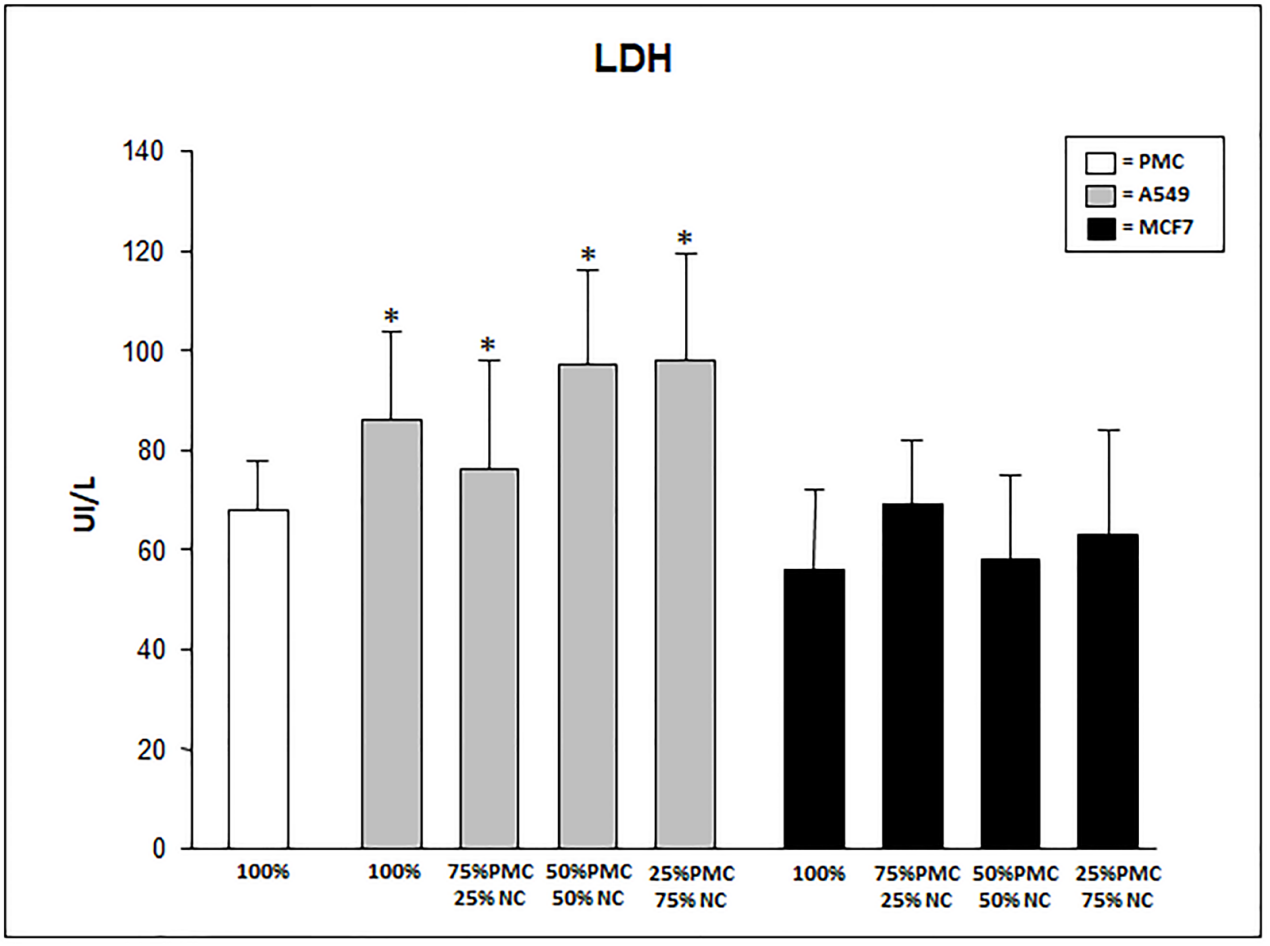
LDH levels produced by talc-exposed PMC, A549 and/or MCF7 cells after 24 hours. PMC = pleural mesothelial cells; NC = neoplastic cells. ^*^ *p* < 0.05 when compared A549 cultures with MCF7 and 100% PMC.

LDH levels were significantly higher in the culture with A549 cells, 100% (86 ± 27) and mixed (87 ± 22, 97 ± 36 and 98 ± 39) when compared with 100% PMC (68 ± 10). In cultures with MCF7 cells (only or mixed), LDH levels were similar to 100% PMC (Figure 2). LDH levels presented about 10% higher in the talc-treated than in untreated cultures.

We did not observe statistical differences in pH measurements comparing treated and untreated groups; however, the highest pH values were observed in 100% PMC (7.45 ± 0.03) with the lowest values in cultures of 100% A549 (7.07 ± 0.03) or 100% MCF7 (7.10 ± 0.10) and 25% PMC + 75% A549 (7.10 ± 0.01) and 25% PMC + 75% MCF7 (7.15 ± 0.13).

### Quantification of viability, necrosis, and apoptosis

Exposure to talc at concentration of 25 μg/cm^2^ reduced the viability of exposed PMC, A549 and/or MCF7 cells compared to unexposed cells in all groups as measured by flow cytometry. The loss of viability could be attributed to both necrosis and apoptosis.

According to these methods, talc induced a significantly higher percentage of cellular necrosis when compared to unexposed cultures, without significant difference between studied groups.

**Figure 3:**
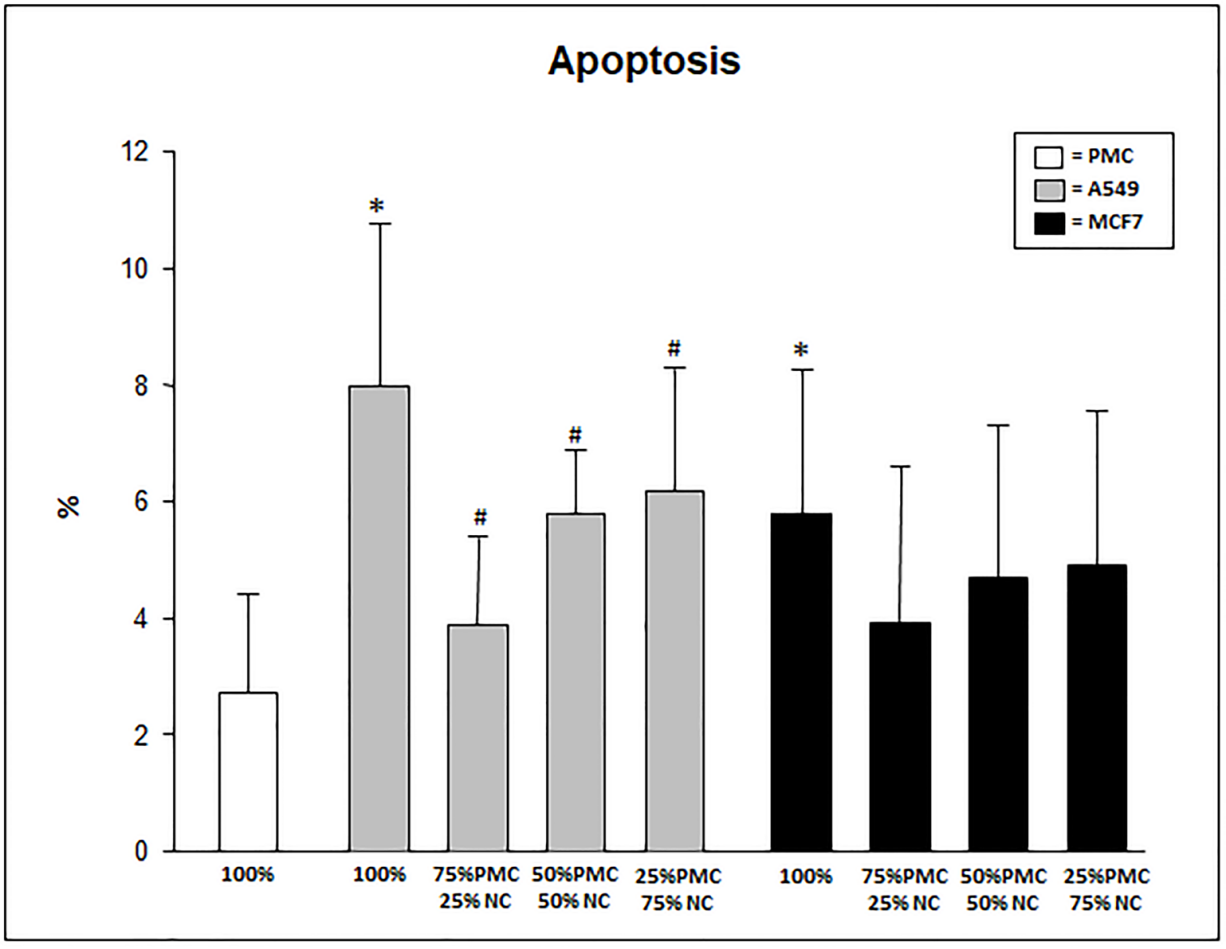
Percentage of apoptosis in PMC, A549 and/or MCF7 after 24 hours exposed to talc. PMC = pleural mesothelial cells; NC = neoplastic cells. ^*^ *p* < 0.05 when compared 100% A549 and MCF7 when 100% PMC; ^#^ *p* < 0.05 when compared 100% A549 when A549 mixed, MCF7 mixed and 100% PMC.

In the evaluation of the percentage of apoptotic cells exposed to the talc particles we can observe significantly higher levels in pure cultures of neoplastic cells than in cultures containing only PMC (*p* < 0.05). The mixed cultures of PMCs and A549 cells showed lower levels of apoptosis in the cultures with fewer PMCs and most cancer cells (*p* < 0.05; Figure 3). The percentage of apoptosis in cultures unexposed to talc was a mean of 1.7 ± 0.7.

## DISCUSSION

Malignant pleural effusion is a common complication in patients with advanced lung or breast cancer and treatment is often performed only as a palliative measure. Talc pleurodesis is a widely used method to prevent recurrence of malignant pleural effusion [2].

In previous *in vivo* and *in vitro studies* using rabbit PMC exposed to talc, the importance of these cells in triggering the acute inflammatory reaction through the production of inflammatory cytokines, was demonstrated [8, 13].

This study demonstrated that mesothelial cells exposed to talc particles for 24 hours produce mediators such as IL-6, IL-1β and TNFRI, recognized in the literature as important mediators of the acute phase of inflammation. In this sense, IL-6 has a particular importance; its function is to influence inflammatory reactions and specific immune responses.

The initial inflammatory process after intrapleural instillation of the sclerosing agent is fundamental for the effectiveness of pleurodesis. In this study cultures containing only NC produced smaller amounts of IL-6, corroborating the findings of Antony and collaborators [5] that demonstrated that in patients with extensive pleural carcinoma and few mesothelial cells, significantly lower levels of fibroblast growth factor (FGF) in the pleural fluid and consequently less chance of success in pleurodesis.

Another proinflammatory cytokine produced by mesothelial cells is the tumor necrosis factor alpha (TNF-α) that directly stimulates the production of other inflammatory mediators. However, this cytokine is extremely sensitive to external interferences such as temperature. In our study, TNF-α levels were undetectable (< 15.6 pg/mL). Some factors may have interfered in these results, such as the low sensitivity of the measurement technique and even the culture medium that does not exactly correspond to the conditions of the pleural cavity *in vivo*.

TNF-α can also act in parallel with TNF receptor I (TNFRI), which has its function linked to apoptosis-inducing proteins and activation of the transcription factor NF-κB [14].

In this study TNFRI presented different behavior compared to the studied neoplastic lineage. In the mixed cultures of mesothelial cells associated with the MCF7 neoplastic lineage, TNFRI indices were proportionally lower in cultures containing the highest neoplastic percentage. In contrast, mixed cultures of mesothelial cells and A549 cells had elevated TNFRI levels. The knowledge in literature about the production of TNFRI by mesothelial and/or neoplastic cells is poorly discussed. In the study using this marker in cachexia-related neoplasms, the selective production of TNFRI was demonstrated and considered by the authors to be important in the inflammatory pathophysiological mechanism in tumors [15].

In this study we investigated the anti-inflammatory cytokine IL-10 because it is described that after the aggression of talc particles in the pleural cavity occurs, an excessive inflammatory stimulus is produced. The PMC attempt to control this inflammation with the production of anti-inflammatory factors such as IL-10. However, its levels were undetectable (< 15.6 pg/mL).

The physical characteristics of talc particles causes in the early stages of pleurodesis, an excessive number necrotic cells that may be characterized by the increase of LDH, an enzyme present in the cytoplasm of different cell types. The detection of LDH is also extremely useful in the laboratory approach to several situations that indicate tissue aggression [10].

In our study LDH showed similar levels in the groups of 100% mesothelial cells and in groups with MCF7 cells. However, we observed higher levels of LDH in the A549 cell groups regardless of the percentage of neoplastic or necrotic cells. LDH production does not only express the physical action of the talc particle with increased necrosis after 24 hours, since there was no difference in the necrosis indices between the groups. These findings demonstrate different behaviors of cell types after exposure to talc.

Another studied biochemical marker in malignant pleural effusion is pH. Heffner et al. [16] reported that patients with pH values below 7.28 had a shorter survival compared to patients with pH above 7.28. Rodriguez-Panadero [11] also observed that the low pH of pleural fluid is correlated with survival and intrapleural tumor progression. The relationship between pleural fluid pH and patient survival may result from the accumulation of glycolysis products in the pleural space caused by extensive tumor deposits, which means an advanced stage or aggressive malignancy with low survival [17]. However, other investigators did not observe an association between pleural fluid pH and tumor involvement [18].

In this study the lowest pH values were observed the in the cultures with neoplastic cells (A549 or MCF7); we have also observed that the use of talc does not interfere with changing pH values.

Apoptosis is a well-studied cell alteration in neoplasms. However, little knowledge about the interaction between normal mesothelial cells and tumor cells is described; several molecules are involved in controlling the pathways of activation of the programmed cell death phenomenon.

Lee and collaborators [12] discussed the importance of cell death mainly caused by apoptosis in mesothelial and/or neoplastic cells leading to the success or failure of pleurodesis, or even action to decrease tumors. In an experimental study it has also been suggested that talc can induce apoptosis in tumor cells and inhibit angiogenesis by presenting a selective action in the mesothelial cells of lower direct aggression, contributing to a better control of malignant pleural effusion [12].

In our study we observed that unlike inflammatory markers, the percentage of apoptosis was higher in cultures of neoplastic cells, especially in A549 cells. This is in agreement with findings of Lee and collaborators [12] and can be explained by the fact that neoplastic cells suffer less inflammation and therefore tend to express lower rates of necrosis and higher rates of apoptosis when submitted to aggression.

These results permit us to infer that the normal mesothelium in contact with the talc particles is the main stimulus in the genesis of the inflammatory process. From the mesothelial activation the production of molecular mediators occurs, and that probably contributes to the dynamics of the local inflammatory process and subsequent production of pleural fibrosis; these mechanisms are necessary to induce effective pleurodesis. These data also allow us to observe that talc has an action in the neoplastic cells inducing higher rates of apoptosis than observed in normal mesothelial cells; this may even contribute in a modest way to tumoral decrease. We can also conclude that different types of tumor cells may respond differently to exposure to talc.

## MATERIALS AND METHODS

This study was approved by the Ethics Committee of the Instituto do Coracao, Hospital das Clinicas, Faculdade de Medicina, Universidade de Sao Paulo, BR (CAPPesq).

Due to pleural mesothelial cells (PMC) being extracted from patients, written informed consent was obtained in accordance with the protocol approved by the institutional review board.

### Cell cultures

Lung adenocarcinoma (A549) and breast adenocarcinoma (MCF7) cells were purchased from the American Type Culture Collection (Manassas, VA).

Human PMC were isolated from pleural fluid obtained via thoracentesis from patients with symptomatic transudative pleural effusion secondary to congestive heart failure and without evidence of infection disease.

All cell lines were cultured at 37°C in 5% CO_2_ –95% air using RPMI culture medium with 10% fetal bovine serum.

### Talc particles

Talc [Mg_6_(OH)_4_(Si2O3)_4_; São Paulo, Brazil] particles were suspended in RPMI culture medium. The median talc particle size was 21.2 μm (range = 6.6–52.6 μm).

### Experimental groups

To mimic different stages of malignant pleural disease we used diverse percentages of mesothelial and neoplastic (A549 or MCF7) cells divided into 5 groups: Group 1: 100% mesothelial cells; Group 2: 100% neoplastic cells; Group 3: 75% mesothelial cells + 25% neoplastic cells; Group 4: 50% mesothelial cells + 50% neoplastic cells and Group 5: 25% mesothelial cells + 75% neoplastic cells.

The groups were exposed to 25 μg/cm^2^ of talc or without talc particles with only RPMI culture medium (control) for 24 hours.

After this period the floating and the adherent cells were collected for determination of viability, necrosis and apoptosis; the supernatant was collected for measurements of cytokines.

### Determination of viability, necrosis, and apoptosis

#### Flow cytometry

Flow cytometry was used to determine the viability, necrosis and apoptosis in cells stained by Annexin V-FITC Apoptosis Detection Kit (BD PharMingen, San Diego, CA, USA) according to the manufacturer’s protocol. Cells stained with annexin V-FITC were considered apoptotic while cells stained with propidium iodide (PI) were considered necrotic; cells not stained with annexin V or PI were considered viable.

### Immunocytochemistry

For detection of apoptotic cells by immunocytochemistry cells were cytospin, placed onto slides and stained for apoptotic DNA fragmentation with TdT FragEL (DNA Fragmentation Detection Kit, Oncogene, Boston, MA) following the manufacturer’s directions. The percent of positively stained nuclei to total nuclei (percent of apoptotic cells) was determined by counting cells with a phase contrast microscope. A total of 1,000 cells were counted per slide and at least three slides per group were examined by two observers. The inter-observer variability was less than 5%.

### Cytokine analysis

IL-6, IL-1β, IL-10, TNF-α and TNF-RI (R&D Systems Inc., Minneapolis, MN, USA) were measured by ELISA (enzyme-linked immunosorbent assay) as described previously [13]. Quantification of cytokines was done by comparison of the optical density in the ELISA reader (Power Wave, Bio-Tek, USA) using a 450 nm filter with the optical density of controls. Minimal detection values for TNF-α, IL-10 and TNF-RI were 15.6 pg/mL, IL-6 was 9.4 pg/mL and IL-1β was 7.8 pg/mL.

### Biochemical assays

LDH was quantified by a kinetic UV method using a commercial kit (Wiener, Argentina) and analyzed in a semi-automatic device. The pH was measured using automated gasometry (Radiometer ABL 800 Flex, Copenhagen, Denmark) in anaerobic conditions.

### Statistical analysis

Data are expressed as mean ± standard deviation. One-way analysis of variance was used to compare difference among groups and the Tukey test was used to perform multiple comparison procedures. Analyses were done using the statistical computer software SigmaStat (Jandel Scientific, San Raphael, CA, USA). A *p* value < 0.05 was accepted as significant.
